# Modelling the dispersal of the two main hosts of the raccoon rabies variant in heterogeneous environments with landscape genetics

**DOI:** 10.1111/eva.12161

**Published:** 2014-05-07

**Authors:** Sébastien Rioux Paquette, Benoit Talbot, Dany Garant, Julien Mainguy, Fanie Pelletier

**Affiliations:** 1 Département de biologie, Université de SherbrookeSherbrooke, QC, Canada; 2 Canada Research Chair in Evolutionary Demography and Conservation, Département de biologie, Université de SherbrookeSherbrooke, QC, Canada; 3 Direction générale de l’expertise sur la faune et ses habitats, Direction de la biodiversité et des maladies de la faune, Ministère du Développement durable, de l’Environnement, de la Faune et des ParcsQuébec, QC, Canada

**Keywords:** dispersal, genetic relatedness, isolation by resistance, *Mephitis mephitis*, multiple regression on distance matrices, *Procyon lotor*, raccoon rabies variant, striped skunk

## Abstract

Predicting the geographic spread of wildlife epidemics requires knowledge about the movement patterns of disease hosts or vectors. The field of landscape genetics provides valuable approaches to study dispersal indirectly, which in turn may be used to understand patterns of disease spread. Here, we applied landscape genetic analyses and spatially explicit models to identify the potential path of raccoon rabies spread in a mesocarnivore community. We used relatedness estimates derived from microsatellite genotypes of raccoons and striped skunks to investigate their dispersal patterns in a heterogeneous landscape composed predominantly of agricultural, forested and residential areas. Samples were collected in an area covering 22 000 km^2^ in southern Québec, where the raccoon rabies variant (RRV) was first detected in 2006. Multiple regressions on distance matrices revealed that genetic distance among male raccoons was strictly a function of geographic distance, while dispersal in female raccoons was significantly reduced by the presence of agricultural fields. In skunks, our results suggested that dispersal is increased in edge habitats between fields and forest fragments in both males and females. Resistance modelling allowed us to identify likely dispersal corridors used by these two rabies hosts, which may prove especially helpful for surveillance and control (e.g. oral vaccination) activities.

## Introduction

Understanding spatiotemporal patterns of pathogen spread is crucial to implement effective actions to contain epidemics (Ostfeld et al. 2005; Vander Wal et al. 2014). Wildlife pathogens, including some that can be very harmful to humans and livestock, are transmitted when infected hosts come in direct or indirect contact with uninfected individuals. In both directly and indirectly transmitted diseases, the extent and speed of propagation is expected to be linked to the dispersal ability of the hosts (Biek and Real 2010). Thus, information about movement and dispersal of hosts is required to identify potential spread pathways. As an example, rivers and highways appear to slow the spread of chronic wasting disease in white-tailed deer (*Odocoileus virginianus*), most likely because they act as barriers to dispersal and gene flow for this species (Blanchong et al. 2008). Similarly, large rivers hamper gene flow in raccoons (*Procyon lotor*) and may reduce the propagation of the raccoon rabies variant (RRV; Cullingham et al. 2009). Control operations that aim at containing and eventually eradicating a given disease are thus likely to be more efficient if positioned alongside these barriers to strengthen their effect (Russell et al. 2006). This strategy was adopted and prevented the northward spread of RRV in Ontario (Canada), in 1999 (Rosatte et al. 2001). Distribution of oral vaccine baits along major rivers to control rabies was performed as early as the 1980s for red foxes (*Vulpes vulpes*), eventually contributing to the elimination of rabies from Switzerland (Wandeler et al. 1988).

Not all environmental barriers to host dispersal and pathogen dissemination, whether natural or of anthropogenic origin, are spatially discrete or easily identifiable, such as rivers and roads, but may instead be continuous or follow a gradient of biotic or abiotic conditions (Storfer et al. 2007). Climate (Geffen et al. 2004), elevation (Shirk et al. 2010) and presence of unsuitable habitats (Goldberg and Waits 2010) are all examples of such limiting conditions. Based on the ecology, behaviour and dispersal ability of host species, these features may restrict pathogen dispersal or promote it through dispersal corridors. Integrating environmental features in models of disease spread can help in predicting the spread and geographic expansion of a disease (Ostfeld et al. 2005).

Different approaches are available to understand the effects of habitat composition on animal movement and dispersal. The first relies on trapping of animals to determine their resource selection and density (Manly et al. 2002), which requires important time and resources to gather large sample sizes. Studies have also been conducted using very high frequency (VHF) transmitters and, more recently, global positioning system (GPS) radio-telemetry to track animal movement and analyse habitat use (Cagnacci et al. 2010). Despite constant technological improvements, collecting large GPS data sets remains very costly and logistically challenging for several species. Spatial simulations can also be used to characterize factors affecting movement and connectivity among individuals in a population (Russell et al. 2006; Rees et al. 2013). While these models can bring insights on the links between habitat and dispersal, the quality of model outputs will depend on an appropriate characterization of ecological processes, which can only be obtained through empirical evaluation. Finally, another approach relies on tools provided by landscape genetics, a discipline integrating aspects of population genetics, landscape ecology and spatial analysis. This field has tremendously progressed in the past 10 years (Manel and Holderegger 2013). Typically, landscape geneticists are interested in describing how gene flow among populations or subpopulations is influenced in often heterogeneous, fragmented landscapes, leading to estimates of functional connectivity (Manel and Holderegger 2013). However, measuring gene flow among groups of individuals imposes limitations on the interpretability of results in terms of functional connectivity because (i) there may be important discrepancies between gene flow and ecological dispersal, that is, movement among habitat patches may not necessarily be associated with opportunities for mating (Garant et al. 2007), and (ii) gene flow measured among populations reflects migration that has occurred for several generations in the past and may not accurately reflect current ecological processes (Epps et al. 2007), including sex-specific differences. Ideally, the operational unit in landscape genetics should be the individual (Manel et al. 2003), in which case estimates of pairwise genetic relatedness can be used as the response variable to model landscape connectivity according to habitat features (Segelbacher et al. 2010; Etherington 2011; Shafer et al. 2012).

Rabies is enzootic to many species of bats and carnivores throughout the world and has a relatively long average incubation period (between 30 and 90 days) in comparison with a short morbidity period (2–10 days) that almost always leads to death (Leung et al. 2007). In eastern North America, the predominant terrestrial rabies strain is the RRV, which has spread in wild populations of both raccoons and striped skunks (*Mephitis mephitis*, hereafter skunks, Guerra et al. 2003). This rabies variant was historically restricted to Florida, but infected raccoons were inadvertently moved to Virginia in the late 1970s and the virus has since expanded northward at a rate of 30–50 km/year (Rupprecht et al. 2002). In Canada, it was first detected in southern Ontario in 1999 (Rosatte et al. 2001), then in New Brunswick in 2000 and finally in Québec in 2006 (Rees et al. 2011). Here, we used estimates of genetic relatedness derived from multilocus microsatellite genotypes to determine which landscape features promoted or limited dispersal of the two main hosts of RRV in an intensively studied area of southern Québec where this viral disease is still under surveillance, control and research activities (Boyer et al. 2011; Houle et al. 2011; Rees et al. 2011; Côté et al. 2012; Mainguy et al. 2012; Talbot et al. 2012).

Previous work in the study area (south-eastern Québec) has shown very little genetic structuring in resident raccoons and skunks, with highways and rivers generally generating a rather weak effect on patterns of genetic differentiation (Côté et al. 2012; Talbot et al. 2012). These equivocal results may conceal the effect of unmeasured spatial variables and do not allow modelling mesocarnivore dispersal at the landscape scale. Our main objective here was to build on the population genetic results obtained in the previous work, using an approach that applies landscape genetic analyses and spatially explicit models, to predict the most likely pathways of skunk and raccoon dispersal and, by extension, terrestrial rabies spread in this area. Based on the ecological knowledge of habitat use by these two hosts, we expected dispersal in both species to be reduced in agricultural fields, but did expect movement to be increased in habitats characterized by a high density of edges (e.g. Glueck et al. 1988; Dijak and Thompson 2000; Larivière and Messier 2000). We expected skunks to be more sensitive to the presence of fields and residential areas than raccoons, as raccoons typically show a greater affinity for dispersal and use cornfields and other human-related food sources (Riley et al. 1998; Prange et al. 2004). We also expected females to be more sensitive to landscape structure than males in both species, as dispersal is usually male-biased in mammals in general (Greenwood 1980), including raccoons and skunks (Cullingham et al. 2008; Côté et al. 2012; Talbot et al. 2012). To our knowledge, this is the first empirical work that addresses movement of these two important rabies hosts in a spatially explicit landscape genetics framework and also the first attempt to quantify the effect of habitat composition on their dispersal. Such work is important to refine predictive models of rabies propagation that use rivers (or other discrete barriers such as mountain chains) and human density indices to predict the rate of propagation of rabies (e.g. Smith et al. 2002; Russell et al. 2005, 2006).

## Materials and methods

### Study area

**Figure 1:**
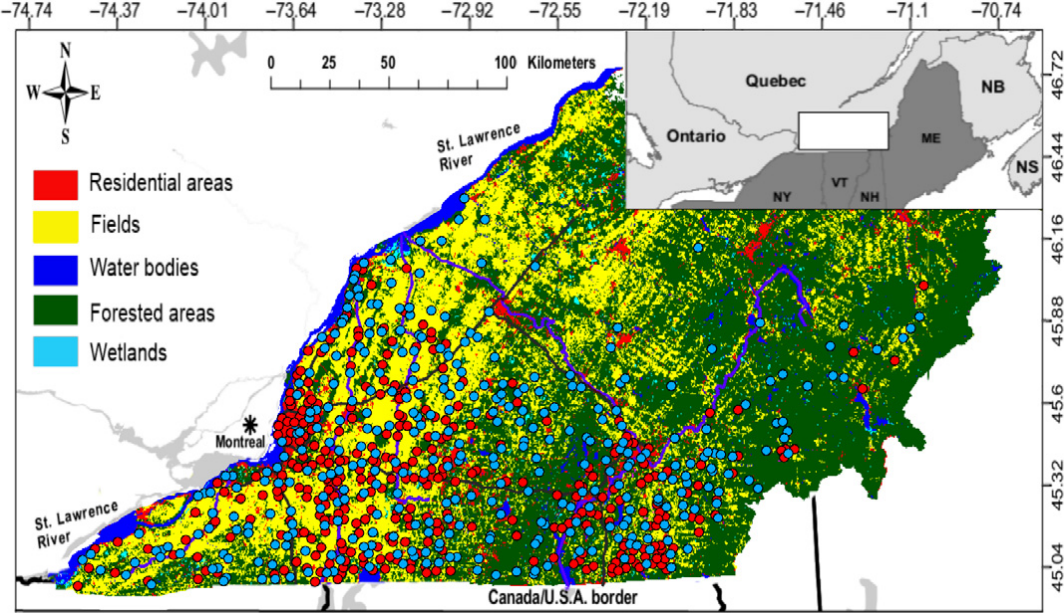
Map of the study area in southern Québec, Canada, which encompasses the RRV monitoring area. Light blue circles indicate the location of sampled raccoons (*n =* 330), and red circles indicate sampled striped skunks (*n =* 345) collected between 2008 and 2010 during rabies surveillance operations.

Our study area was located in southern Québec, Canada (45°23′ N, 72°43′ W), where all known positive cases of rabies in the province of Québec have been recorded between 2006 and 2009 (Fig. 1). We used biological samples collected on raccoons and skunks over 3 years (2008–2010) in this RRV epizootic region, over an area of approximately 22 000 km^2^ (Fig. 1). This area corresponds to the so-called RRV monitoring area where rabies-related surveillance has been increased since 2006. From east to west, the study area follows a gradient of increasing agricultural intensification and urbanization (Ghilain and Bélisle 2008) where hayfields and pastures in the east are gradually replaced by large-scale, continuous row cropping for corn, cereals and soyabean. Forest cover also follows this gradient, as it becomes smaller and more fragmented along the gradient of agricultural intensification.

### Sampling

Tissue samples used in this study were collected by the Québec Ministère des Ressources Naturelles et de la Faune (MRNF) and its partners between 2008 and 2010 during surveillance and control activities (see Rees et al. 2011; Côté et al. 2012; Talbot et al. 2012). They included samples either taken from recovered road-killed animals (Rees et al. 2011) or collected during post-oral rabies vaccination campaigns (Mainguy et al. 2012). All samples were georeferenced using a hand-held GPS device. A skin biopsy was collected from the ear with a 2-mm punch for genetic analyses. Samples were stored in 95% ethanol until DNA extraction. Because many animals were sampled over small areas due to field activities conducted in specific zones (≈ 20 individuals/100 km^2^ zones), a random subset of individuals (up to 3 individuals/25 km^2^) was selected for each year to generate a sample as evenly distributed as possible over the study area (Fig. 1). A total of 330 raccoon samples (192 males and 138 females) and 345 striped skunk samples (195 males and 150 females) were retained for this study. There was no geographic bias in the locations of samples for both sexes.

### Genetic analyses

DNA extraction was conducted using a protocol described in Chambers and Garant (2010). Microsatellite polymorphism was assessed at ten loci developed for raccoons (see Côté et al. 2012 for the list of loci used and PCR protocols) and at nine loci developed for skunks (see Talbot et al. 2012). DNA amplification was performed using geneamp system 9700 thermocyclers (Applied Biosystems, Foster City, CA, USA). Genotyping was performed using an AB 3130 DNA sequencer (Applied Biosystems), and allele size was scored using genemapper 4.0 (Applied Biosystems). All microsatellite loci were tested for departures from Hardy–Weinberg equilibrium and linkage disequilibrium with a sequential Bonferroni correction, and indices of genetic diversity (number of alleles and observed and expected heterozygosity) were computed using genepop 4.0 (Raymond and Rousset 1995) and popgenkit (Rioux Paquette 2011). Finally, molecular sexing was conducted using a modification of Shaw et al. (2003) as described in Côté et al. (2012).

### Landscape genetics

We applied two types of analyses that both aim at finding the model that best explains patterns of genetic distances among individuals. The first approach (multiple regression on distance matrices or MRM; Legendre et al. 1994; Lichstein 2007) provides a way to statistically test the influence of a set of variables (e.g. geographic distance and land cover types) on pairwise genetic distances in a regression model and retain only those that have a significant effect. The second approach (isolation-by-resistance models or IBR, McRae 2006) is based on circuit theory and treats the landscape as a surface with various electrical resistances, while dispersing individuals (or gene flow) are analogous to electrical current. Any number of models with different landscape resistances can be tested, but we restricted their number to a small number of competing hypotheses that integrated results from MRM. The model that best fits genetic distances can further be used to illustrate dispersal at the landscape scale. The following paragraphs describe these statistical analyses in details.

#### Calculation of genetic distance

An estimator of pairwise genetic relatedness (*r*_xy_; Wang 2002) was calculated using spagedi 1.3 (Hardy and Vekemans 2002). We converted relatedness coefficients to genetic distances using 1 – *r*_xy_ for easier interpretation. In all analyses, these were used as an index of genetic dissimilarity among all pairs of sampled individuals.

#### Multiple regression on distance matrices

MRM is derived from partial Mantel tests of matrix correlations, in which predictor matrices (in this case, landscape variables, see paragraph below and Appendix A) are used to explain variation in a response matrix (genetic distance). Because of nonindependence of observations in pairwise distance matrices, MRM comprises a permutation procedure that takes into account the structure of distance matrices (i.e. keeping observations belonging to a given individual together) to assess the statistical significance of explanatory variables (Legendre et al. 1994).

To generate landscape variables, southern Québec was mapped using aerial photographs provided by the MRNF (Fig. 1) in arcgis 9.2 (Environmental Systems Research Institute, Redlands, CA, USA). Landscape was characterized by assigning each pixel to one of five categories of land type (see Table 1). We selected these 5 habitat categories based on a previous study on raccoon habitat conducted by Houle et al. (2011) in this region. A buffer zone was traced around each pair of individuals, within each species. The length of the buffer was equal to the Euclidian distance between the two individuals (ranging from 0 to 305 km; mean ± SD: 84.0 ± 47.4 km). The width of the buffer was equal to 4 km. This arbitrary value was selected as a reasonable width since the home range of raccoons in rural Ontario is typically < 4 km^2^ (Rosatte 2000). An illustration of the buffer tracing method is provided in Appendix A for clarity. Landscape composition (percentage of a given buffer covered by each habitat type) was calculated using hawth’s tools (Beyer 2004) in arcgis 9.2. The length of edges between forested and agricultural areas (fields) was calculated within each of the buffer zones, also using hawth’s tools, and divided by the area of the buffer zone, to obtain the edge density separating each pair of individuals of the study. In addition to the continuous landscape variables, major rivers and highways (i.e. landscape barriers) were also included in the analyses. These barriers were also mapped with aerial photographs provided by the MRNF using arcgis 9.2. We selected rivers that are known to maintain an important discharge throughout the year (water flow > 0.6 m/s, as in Talbot et al. (2012): Richelieu, St. François, Châteauguay, Yamaska and Magog rivers). We selected highways based on high speed limit (100 km/h) and the absence of crossroads. We calculated how many times the Euclidian distance between each pair of samples intersected with a section of a major river or a highway using hawth’s tools.

**Table 1:** Description of continuous landscape variables included in this study, along with the percentage of the landscape they covered and their range in MRM pairwise buffers (see Materials and methods).

Continuous landscape variables	Description	Proportion of the landscape (%)	Range in MRM buffers
Landscape composition			
Field proportion (%)	Natural open areas and cropfields	46.0	[0.000–1.000]
Forested lands’ proportion (%)	Natural forests, logged and sylviculture areas	44.1	[0.000–0.994]
Wetlands’ proportion (%)	Bogs, fens and swamps	0.4	[0.000–0.249]
Open water proportion (%)	Rivers and lakes	2.2	[0.000–0.615]
Residential area proportion (%)	Urban agglomerations and areas dominated by human infrastructures	7.2	[0.000–0.889]
Landscape structure			
Edge density (km/km^2^)	Edges between parcels of open fields and forested lands	–	[0.000–10.972]

MRM models were computed for the two species when including all individuals and then separately for both sexes using the R package ecodist v. 1.2.2 (Goslee and Urban 2007), and statistical significance was assessed with 10 000 permutations in each case. Standard errors on model coefficients were estimated by jackknifing individual data. Initial MRM models included geographic distance (km), number of major river crossings, number of highway crossings, proportion of fields, proportion of residential areas, proportion of wetlands and edge density. A backward selection procedure was then applied (*P =* 0.05) to progressively eliminate nonsignificant variables from the models. While the suitability of stepwise methods (including backward elimination) to select regression variables has been debated (see Whittingham et al. 2006; but Murtaugh 2009 for a counterargument in support of their use), we mainly chose this approach because common alternatives (e.g. comparing models on the basis of Akaike information criterion [AIC] values) cannot be applied in a dissimilarity framework in which independence of observations is not respected. This is a common issue in landscape genetics because pairwise genetic distances may not be independent (Goldberg and Waits 2010). The proportion of forested areas was not included in the models, as it was very strongly correlated (negatively) with the proportion of agriculture areas (*r* < −0.9) and explained a smaller proportion of the variance. The proportion of open water was also excluded because the number of rivers was already included as a discrete landscape variable.

#### Isolation-by-resistance models

In the past, a large number of studies have relied solely on Euclidian distance (isolation-by-distance model, or IBD) or on the number of discrete barriers (isolation-by-barrier model, or IBB) between samples to explain genetic differentiation. However, the IBR framework (McRae 2006) has recently emerged as a valuable approach in landscape genetics to assess ‘effective distance’, that is, the actual distance an individual would need to travel between two points, assuming he chooses a path of least resistance (Amos et al. 2012). After assigning a resistance value to every element of the landscape, an algorithm computes resistance distances between pairs of points on the surface. In several cases, resistance distances explain patterns of genetic differentiation much more accurately than regular Euclidian distances (e.g. McRae and Beier 2007).

We used circuitscape (McRae and Shah 2009) to compute pairwise resistance distances. This program uses a raster file (map) and a list of coordinates (sampling points) as input. Seven competing models of landscape resistance, each characterized by specific resistance values assigned to landscape elements, were evaluated (see Table 2 for assigned values). These models reflected hypotheses of IBD, IBB and IBR that were plausible in the light of MRM results. The seven corresponding raster files were prepared from the map described above using QGIS (QGIS Development Team 2013, Open Source Geospatial Foundation Project) and the R package SDMTools (VanDerWal et al. 2012).

**Table 2:** Resistance values used for landscape elements included in the seven different isolation-by-resistance (IBR) models tested to explain patterns of genetic distance among individual raccoons and skunks.

	Forest–field edges	Forested areas and wetlands	Residential areas	Fields	Highways	Rivers and water bodies
Model 1	50	50	75	100	1000	5000
Model 2	50	50	500	5000	50 000	500 000
Model 3	50	50	75	100	100	5000
Model 4	50	50	75	100	100	100
Model 5*	50	50	50	50	1000	5000
Model 6†	50	50	50	50	50	50
Model 7	10	50	75	100	100	100

* Corresponds to a model of isolation by barriers (IBB).

† Corresponds to a model of isolation by distance (IBD).

After running circuitscape and obtaining pairwise resistance distances for each model, they were compared by fitting linear models regressing genetic distance against resistance distance (i.e. IBR models), with R version 3.0.1 (R Development Core Team 2013). Because of nonindependence of pairwise distances, we could not rely on the use of an information theoretic method (e.g. AIC values) to compare models. Instead, we applied the pseudo-bootstrap approach of Worthington Wilmer et al. (2008) to select the best IBR model. This approach consists in retaining, for each pseudo-bootstrap replicate, only a randomly chosen subset of distance values that are completely independent from one another. For instance, for our data set of 330 raccoons, a maximum of 165 independent distance values can be obtained. For each replicate, linear models for the different resistance models are fitted and the one with the lowest AIC is selected as the best model. After a large number of replicates, the model most often selected is considered the best IBR model. In our case, 50 000 pseudo-bootstrap replicates were performed for each species and each sex within species with a custom-made R script (available on request from the authors). The best models were used to produce maps of landscape resistance to visualize zones of high dispersal (i.e. corridors) and zones of high resistance across the study area.

## Results

For the two species, no pair of loci exhibited significant linkage disequilibrium. A single locus showed a significant departure from Hardy–Weinberg equilibrium (locus PLM20 in raccoons). All loci were highly variable: the number of alleles per locus in raccoons ranged from 5 to 26, *H*_O_ between 0.685 and 0.888, and *H*_E_ between 0.754 and 0.931. In skunks, the number of alleles per locus ranged from 7 to 15, while *H*_O_ varied between 0.588 and 0.881 and *H*_E_ between 0.620 and 0.896. Polymorphism information for all loci is provided in Appendix B. These summary results indicated that the microsatellite data sets were suitable for further analyses.

### MRM analyses

Globally, MRM models showed that raccoon dispersal was generally less sensitive to landscape composition than in skunks. These models also showed that females of both species were more sensitive to landscape structure than males. All results from MRM models, including the final models retained after backward selection of variables, are reported in Table 3. In raccoons, the analysis including all individuals and the one restricted to males showed that the only significant predictor of genetic distance among individuals was geographic distance. In females, the only significant variable in the final model was the proportion of agricultural fields, as genetic distance among females increased with the proportion of fields in the landscape. In skunks, for models computed either with all individuals or with females only, genetic distance increased with geographic distance and proportion of agricultural fields, but decreased as the amount of forest edges increased in the landscape (Table 3). In contrast, male skunks did not exhibit detectable sensitivity to agriculture.

**Table 3:** Values of MRM regression coefficients (*β)* in models explaining species-specific genetic distance among individual raccoons or skunks. For both species, analyses were conducted with all individuals at first and then separately for males and females. For each analysis, the complete regression model is shown (with all variables), as well as the final model resulting from backward elimination of nonsignificant variables.

	Explanatory variable	*β*	SE	*P-*value
Raccoons (all individuals (*n* = 330))	Geographic distance (km)	1.16 × 10^−4^	8.14 × 10^−1^	0.089
	Number of rivers	−3.83 × 10^−3^	2.66 × 10^−3^	0.104
	Number of highways	3.56 × 10^−3^	2.38 × 10^−3^	0.081
	% Fields	9.85 × 10^−3^	1.29 × 10^−2^	0.453
	% Residential land	−4.27 × 10^−2^	3.45 × 10^−2^	0.215
	% Wetlands	7.14 × 10^−2^	2.51 × 10^−1^	0.820
	Edge density (km/km^2^)	2.57 × 10^−5^	2.53 × 10^−3^	0.992
Final model	Geographic distance (km)	**9.59 × 10**^**−5**^	**4.69 × 10**^**−5**^	**0.011**
Raccoons (males (*n* = 192))	Geographic distance (km)	1.41 × 10^−4^	1.30 × 10^−4^	0.153
	Number of rivers	−3.50 × 10^−3^	4.03 × 10^−3^	0.284
	Number of highways	2.98 × 10^−3^	3.18 × 10^−3^	0.297
	% Fields	4.41 × 10^−3^	1.90 × 10^−2^	0.812
	% Residential land	−7.50 × 10^−2^	4.26 × 10^−2^	0.105
	% Wetlands	5.88 × 10^−2^	2.79 × 10^−1^	0.897
	Edge density (km/km^2^)	−2.95 × 10^−3^	5.03 × 10^−3^	0.355
Final model	Geographic distance (km)	**1.33 × 10**^**−4**^	**7.24 × 10**^**−5**^	**0.010**
Raccoons (females (*n* = 138))	Geographic distance (km)	9.71 × 10^−4^	1.16 × 10^−4^	0.140
	Number of rivers	−5.94 × 10^−3^	4.09 × 10^−3^	0.115
	Number of highways	4.94 × 10^−3^	4.27 × 10^−3^	0.164
	% Fields	**4.33 × 10**^**−2**^	**2.06 × 10**^**−2**^	**0.022**
	% Residential land	2.64 × 10^−2^	7.25 × 10^−2^	0.672
	% Wetlands	−3.04 × 10^−1^	7.44 × 10^−1^	0.552
	Edge density (km/km^2^)	3.17 × 10^−3^	5.28 × 10^−3^	0.424
Final model	% Fields	**3.95 × 10**^**−2**^	**1.77 × 10**^**−2**^	**0.028**
Skunks (all individuals (*n* = 345))	Geographic distance (km)	**3.45 × 10**^**−4**^	**1.12 × 10**^**−4**^	**0.003**
	Number of rivers	2.29 × 10^−3^	4.26 × 10^−3^	0.504
	Number of highways	3.36 × 10^−3^	3.17 × 10^−3^	0.249
	% Fields	**6.20 × 10**^**−2**^	**2.14 × 10**^**−2**^	**0.001**
	% Residential land	7.34 × 10^−2^	5.41 × 10^−2^	0.102
	% Wetlands	5.46 × 10^−1^	4.07 × 10^−1^	0.184
	Edge density (km/km^2^)	**−2.36 × 10**^**−2**^	**1.15 × 10**^**−3**^	**0.033**
Final model	Geographic distance (km)	**3.86 × 10**^**−4**^	**6.25 × 10**^**−5**^	**<0.001**
	% Fields	**5.09 × 10**^**−2**^	**1.80 × 10**^**−2**^	**0.001**
	Edge density (km/km^2^)	**−4.05 × 10**^**−2**^	**1.04 × 10**^**−2**^	**<0.001**
Skunks (males (*n* = 195))	Geographic distance (km)	2.92 × 10^−4^	1.77 × 10^−4^	0.064
	Number of rivers	4.38 × 10^−3^	6.32 × 10^−3^	0.375
	Number of highways	3.84 × 10^−3^	4.82 × 10^−3^	0.364
	% Fields	4.13 × 10^−2^	3.03 × 10^−2^	0.138
	% Residential land	2.70 × 10^−2^	7.28 × 10^−2^	0.671
	% Wetlands	8.49 × 10^−1^	6.36 × 10^−1^	0.154
	Edge density (km/km^2^)	−2.25 × 10^−2^	1.72 × 10^−2^	0.155
Final model	Geographic distance (km)	**3.39 × 10**^**−4**^	**8.39 × 10**^**−5**^	**<0.001**
	Edge density (km/km^2^)	**−4.12 × 10**^**−2**^	**1.60 × 10**^**−2**^	**0.005**
Skunks (females (*n* = 150))	Geographic distance (km)	**3.47 × 10**^**−4**^	**1.93 × 10**^**−4**^	**0.029**
	Number of rivers	1.65 × 10^−3^	6.58 × 10^−3^	0.757
	Number of highways	2.83 × 10^−3^	4.65 × 10^−3^	0.495
	% Fields	**7.37 × 10**^**−2**^	**3.09 × 10**^**−2**^	**0.007**
	% Residential land	**1.31 × 10**^**−1**^	**8.15 × 10**^**−2**^	**0.038**
	% Wetlands	−1.83 × 10^−1^	4.80 × 10^−1^	0.767
	Edge density (km/km^2^)	−2.65 × 10^−2^	1.39 × 10^−2^	0.087
Final model	Geographic distance (km)	**3.44 × 10**^**−4**^	**9.48 × 10**^**−5**^	**<0.001**
	% Fields	**5.83 × 10**^**−2**^	**2.59 × 10**^**−2**^	**0.010**
	Edge density (km/km^2^)	**−5.17 × 10**^**−2**^	**1.36 × 10**^**−2**^	**<0.001**

Standard errors (SE) and *P-*values estimated from 10 000 permutations are provided. Significant results (*P* < 0.05) are indicated in bold.

### Isolation-by-resistance models

**Figure 2:**
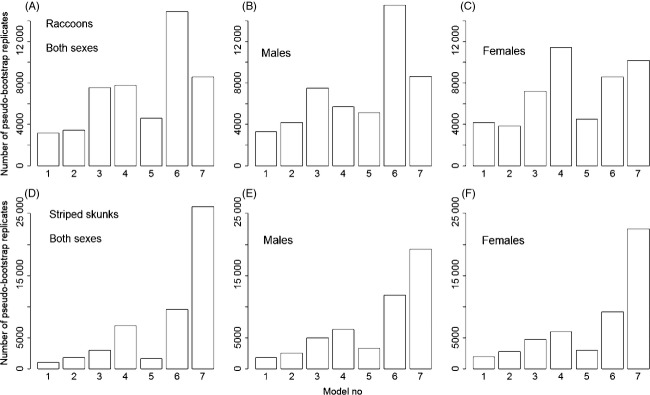
Distribution of pseudo-bootstrap replicates according to which model of isolation by resistance (IBR) had the lowest Akaike information criterion (AIC) values; among seven different models considered (see Table 2 for landscape resistance values of all models), IBR models were fitted to explain genetic distances among raccoons (A–C) and striped skunks (D–F), first by combining data from both sexes and then by analysing each sex separately. A total of 50 000 pseudo-bootstrap replicates were performed for each analysis.

In raccoons, the pseudo-bootstrap procedure revealed the greatest support for a simple model of isolation by distance (model 6 from Table 2), both when considering all individuals or males only (Fig. 2). In females, model 4 was best supported, indicating that agricultural fields show a greater resistance to dispersal than forested patches or residential areas. However, support for models 6 (IBD) and 7 (edge effect) was only slightly lower in female raccoons, and support for model 7 was found to in fact surpass that of model 4 when edge resistance was fitted as being only 1/50th of forest resistance (see Appendix C). In skunks, all three set of analyses [(i) all individuals, (ii) males and (iii) females] indicated the greatest support for model 7 (Fig. 2), the most complex model of landscape resistance considered (edges with the lowest resistance, followed by forest fragments, residential areas and then fields; Table 2). For both species, none of the selected models included highways or rivers as greater barriers to dispersal than agricultural fields.

**Figure 3:**
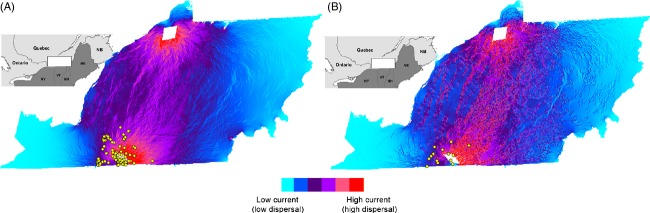
Current maps illustrating likely dispersal paths from the last recorded case of RRV-positive animals in the southern portion of our study site to the northern part of our study site. In these maps, the landscape is analogous to a surface with various electrical resistances, and dispersal is analogous to electrical current avoiding high resistances from one point to the other. Panel (A) illustrates dispersal of female raccoons, while (B) illustrates dispersal of striped skunks (either males or females). Yellow dots indicate the location of the 88 RRV-positive cases recorded in raccoons (A) and 14 cases in striped skunks (B) between 2006 and 2009 for the province of Québec.

Using resistance values from the selected models for each species and sex combination, we generated current maps, defined as such because of the analogy with electrical resistance (McRae and Shah 2009), illustrating the hypothetical paths of a raccoon or skunk dispersing from the last known record of rabies in Québec to the northernmost boundary of our study site. Figure 3A shows the case of a female raccoon, while Fig. 3B represents the same hypothesis for a skunk (either male or female). The male raccoon scenario is not shown because a simple model of IBD best explained male raccoon dispersal. On these maps, dispersal corridors are visible from south to north in the central part of the study area, which is associated with the presence of forest patches in an otherwise agriculturally dominated landscape.

## Discussion

In this study, we used a combination of MRM and resistance analyses to identify landscape features affecting dispersal in raccoons and striped skunks and to predict the most likely pathways of terrestrial rabies spread by these hosts. As expected, we found that raccoon dispersal was generally less sensitive to landscape composition than in skunks and that females of both species were more sensitive to landscape structure than males. Our results also suggest that the most likely pathways for northward host dispersal in the region are in the centre or our study area in corridors associated with forested fragments where the landscape sharply shifts from predominantly extensive farming to mostly intensive agriculture. Previous studies have shown no difference in dispersal distance or general behaviour between rabies-infected and other individuals in raccoons (Rosatte et al. 2006) and skunks (Greenwood et al. 1997) and no effect of the presence of rabid individuals on the resulting genetic population structure (Talbot et al. 2014). Thus, we argue that modelling the dispersal of hosts in heterogeneous landscapes can, by extension, allow a better understanding and forecasting of rabies spread, especially when combined with ecological epidemiological models (e.g. Russell et al. 2005; Rees et al. 2013).

### Dispersal in raccoons and striped skunks

Our initial prediction that dispersal in raccoons would be less sensitive to landscape heterogeneity than in skunks was supported globally, and other studies support frequent long-distance dispersal in raccoons. For instance, 10% of natal dispersal distances were >10 km in southern Ontario (Cullingham et al. 2008), whereas in an agriculturally fragmented landscape in Indiana (USA), long-distance dispersers accounted for 10% of the population and there was no pattern of IBD among habitat patches (Dharmarajan et al. 2009). In the similarly heterogeneous landscape of our study site, geographic distance was the main predictor of pairwise relatedness in raccoons. This indicates that intrinsic raccoon behaviour and propensity to disperse are more important than landscape composition in shaping patterns of interindividual genetic distance at the scale of the study. The only significant landscape effect was observed in female raccoons, for which an increase in the proportion of agricultural fields for a given area leads to an increase in pairwise genetic distances. This result is suggestive of reduced dispersal in that habitat type. In raccoons, females are the most philopatric sex (Cullingham et al. 2008; Côté et al. 2012), and thus, we expected dispersal to be more affected by landscape resistance in this sex. Our results also help explaining the results of Dharmarajan et al. (2009), who found that in an agricultural landscape, 50% of males dispersed over distances larger than 5 km, whereas this proportion was only 19% in females. Further support for this comes from a radio-telemetry study conducted by Beasley and Rhodes (2010) that indicated that female raccoons, as opposed to males, tend to concentrate their activities in remnant forested patches within agriculturally dominated areas.

Interpreting landscape resistance in terms of habitat quality can be misleading without field-based ecological evidence, because dispersal is influenced by individual and population conditions (Ronce et al. 2001). For instance, a high-quality habitat may reduce dispersal if all resources needed by an organism are found within a small area, but may stimulate dispersal if a habitat patch becomes overcrowded. Indeed, dispersal in raccoons is often considered to be partly driven through a density-dependent process (Russell et al. 2006), and local patch dynamics may explain patterns of dispersal in fragmented landscapes (Dharmarajan et al. 2009). In the case of the effect of agricultural fields on female raccoon dispersal, field studies of raccoon habitat use have suggested that raccoons generally avoid field interiors (Fritzell 1978; Glueck et al. 1988; Beasley and Rhodes 2010). This would support the idea that agricultural lands reduce raccoon dispersal because they represent low-quality habitat. Conversely, edges between forest patches and fields may still represent particularly suitable habitat for raccoons (Dijak and Thompson 2000; Barding and Nelson 2008), where they tend to be found in higher abundance when compared to agriculturally dominated areas, as previously documented in our study area (Houle et al. 2011).

In skunks, the model of greater dispersal in habitat edges received the strongest support in all three analyses (all individuals combined, males and females). We expected skunks to be more sensitive to landscape heterogeneity based on previous studies of their habitat use that emphasized the preference of skunks for ecotones (Larivière and Messier 2000; Frey and Conover 2006). While it is interesting that this preference was captured in our IBR analyses derived from microsatellite-based relatedness, its impact on the resulting current maps is rather weak, as modelling of dispersal across the landscape is very similar for skunks and female raccoons (Fig. 3A,B). These maps emphasize that, globally, raccoons and skunks respond similarly to landscape heterogeneity, and the IBR model of edge effects (no. 7, i.e. the best model in skunks) was the second-best model in all three raccoon analyses (Fig. 2A–C), especially in females (see Appendix C also). This reinforces the proposed idea that control operations against RRV spread should particularly target areas where agricultural fields and forest patches are greatly interspersed (Boyer et al. 2011; Houle et al. 2011).

Perhaps one of the most surprising results of our study is the lack of support for rivers and highways as barriers to dispersal within our study area. Previous population genetic work covering the same geographic area suggested that in both species, rivers significantly constrained gene flow, albeit weakly (Côté et al. 2012; Talbot et al. 2012). These analyses did not include landscape composition data. We found no evidence that rivers and highways act as greater barriers to dispersal than agricultural fields when accounting for landscape heterogeneity. This discrepancy emphasizes the need for caution when interpreting tests of IBD or IBB, as collinearity between either geographic distance or the number of rivers and other missing or excluded landscape variables may lead to spurious conclusions about the real processes driving spatial genetic structure (Cushman and Landguth 2010). Additionally, rivers that were included as possible barriers were selected based on their mean discharge, but it is still possible that some sections are much easier to cross than others, which could weaken the signal for their effect as natural barriers.

### Value of combining MRM and resistance analyses

The approach we used in this work allowed us to describe the dispersal of two terrestrial vectors of rabies living in a heterogeneous landscape from pairwise relatedness estimates (converted to distance) among sampled individuals. Analyses of processes driving spatial genetic structure are only reliable if these processes are sufficiently homogeneous at the scale of the study area (Rioux Paquette et al. 2010; Segelbacher et al. 2010). For this reason, it is necessary to ensure that the studied populations represent single, genetically homogeneous clusters (Born et al. 2008). Through the use of Bayesian clustering algorithms, this verification has already been carried out, for both the raccoon and the skunk populations of the RRV monitoring area in southern Québec (Côté et al. 2012; Talbot et al. 2012, 2014). Although microsatellite data can lead to somewhat imprecise estimates of pairwise relatedness (Van de Casteele et al. 2001; Csilléri et al. 2006), they should still reflect ‘true’ genetic relatedness (e.g. Mainguy et al. 2009) and thus should allow detecting species- and sex-specific dispersal patterns without relying on a very large data set. We believe that this makes our approach a valuable tool for future investigations of possible pathways of dispersion in the context of disease management.

Concerns about the statistical power and biases related to the application of the partial Mantel test and its derived forms (e.g. MRM) have been raised (Legendre and Fortin 2010; Guillot and Rousset 2013). Nonetheless, in landscape genetics, the problem often lies in choosing among several statistically supported models, in which case MRM may be especially helpful (Wagner and Fortin 2013). Simulation studies indeed suggest that partial Mantel tests and causal modelling with MRM are reliable to distinguish hypotheses of IBD, IBB or IBR (e.g. Balkenhol et al. 2009; Rioux Paquette and Lapointe 2009; Cushman and Landguth 2010; but see Cushman et al. 2013). A possible limitation of the method is the potential sensitivity of the results to different sizes of geographic buffers around pairs of sampled individuals. However, we evaluated this possibility and found that habitat composition values were highly correlated for different buffer sizes (e.g. correlations of values obtained for 2-km vs 4-km buffers were 0.940 and 0.936 for forest and fields, respectively, while they were 0.853 and 0.820 for 4-km vs 8-km buffers and remained above 0.750 until buffers reached 20 km). Thus, results should not be very sensitive to variations within this range of buffers. Here, we mostly used MRM to provide an empirical evaluation of the effect of various landscape elements on dispersal to restrict the number of considered IBR models to a small number. Expert opinion models of landscape resistance are often subjective and can be misleading (Shirk et al. 2010; Spear et al. 2010), so relying on empirical evidence to establish a list of hypothesis-based models is advised (Spear et al. 2010). It is possible to perform optimization procedures to obtain the resistance values that maximize the fit between genetic and resistance distances (Shirk et al. 2010). However, the calculation of IBR models might be computationally intensive, especially when using individuals as the sampling unit rather than populations, which increases the data set size. Furthermore, optimizing the fit of resistance and individual genetic distances would probably lead to overfitting considering the uncertainty associated with pairwise relatedness estimates derived from a relatively small number of markers (Van de Casteele et al. 2001) and knowing that the relative importance of landscape features may vary in different areas (Short Bull et al. 2011). Our objective was to compare the support of a restricted set of possible hypotheses regarding landscape resistance, but different resistance values may affect results (Spear et al. 2010). Most notably, in the case of female raccoons, for which IBR results were equivocal, lowering the value of edge resistance to 2% of the forest resistance (instead of 20% as in Table 1) leads to that model receiving slightly greater bootstrap support than the one that was previously best supported (Appendix C). In all other cases where one model clearly had greater support, results were not sensitive to edge resistance variation (Appendix C). Final results from IBR analyses and those from MRM were globally consistent.

Finally, the pseudo-bootstrap method of Worthington Wilmer et al. (2008) that we used for selection of IBR models provided a simple solution to the issue of inflated sample sizes when working with distance matrices (Wagner and Fortin 2013). It also provides an information theoretic method (i.e. using AIC) to select among competing IBR models without relying on partial Mantel tests between resistance and genetic distances, which can be problematic because of very high autocorrelation among IBR models (Cushman et al. 2013).

### Implications on rabies surveillance and control

Most researchers who have investigated raccoon dispersal patterns have found that major rivers represent key semipermeable barriers (e.g. Smith et al. 2002; Cullingham et al. 2009). These provide ideal opportunities for efficient oral vaccination campaigns through reinforcement of natural barriers (Wandeler et al. 1988; Rosatte et al. 2001). Our landscape genetic results show that in this case, models accounting for landscape composition perform better than those that only include discrete barriers and provide further insights about previous population genetics work that had revealed weak effects of rivers on the gene flow of raccoons and skunks in the RRV monitoring zone at the same geographic scale (Côté et al. 2012; Talbot et al. 2012). Depending on the study area, additional landscape features may influence dispersal, such as ‘landscape shape’, for example, a terrestrial constriction between water bodies (Rees et al. 2009). At the landscape scale, being able to accurately model host dispersal is required to develop appropriate surveillance and/or control strategies, including the distribution of oral vaccine baits (e.g. Boyer et al. 2011). Our approach has allowed us to identify potential dispersal corridors between the last known rabies-positive cases close to the US–Canada border and the north of the RRV monitoring area. These corridors could be targeted as areas of high importance should RRV reach southern Québec again. It is important to note that the current maps illustrate resistance between the source (the location where rabies cases have been detected) and ground node, which we chose, in this case, to place at the north end of the study area, considering the northward trend in RRV expansion. If the ground node was modified (e.g. if we wished to model dispersal to the north-eastern boundary of the map on Fig. 3), the resulting current map would change, illustrating the paths of least resistance in that direction. Recent developments of the circuitscape model may allow visual representation of connectivity in all directions across the landscape (Pelletier et al. 2014), so this limitation could be avoided depending on the research questions. In addition, landscape resistance values for the different habitat types could be incorporated in models of rabies propagation that already account for discrete barriers and human density (e.g. Russell et al. 2005, 2006). It would be especially interesting to combine our findings with the recent simulation work of Rees et al. (2013), which is the first to integrate habitat quality and heterogeneity in spatial simulations of rabies vaccination efficacy. Models combining landscape genetics with ecological epidemiology could be used to determine the most likely path of disease spread, at the landscape scale, between the location of a documented positive case and one or many nearby cities to assess, among other things, health risks for human populations.

Following the first RRV-positive case recorded in the province in 2006, the control campaigns performed by the Québec MRNF in the past years have been effective: since 2009, there have been no reported cases despite the maintenance of intensive surveillance operations (Rees et al. 2011). Nevertheless, considering that 218 rabid raccoons and 88 rabid skunks we recorded in the four US states sharing a border with Québec in 2011 alone (Blanton et al. 2012), the province likely will continue to be vulnerable to the possibility of receiving dispersing rabid animals. There are no rivers or other discrete barriers to dispersal along the US–Québec border; consequently, applying resistance models that integrate habitat composition to model dispersal pathways, like the ones we presented here, may enhance current operations aimed at preventing RRV from re-entering the province. We believe this is especially relevant in the light of analyses showing that the economic efficiency of the provincial rabies management programme in the future will mainly depend on minimizing programme costs (Shwiff et al. 2013).

## Data archiving statement

The data sets used in this work have been uploaded to the Dryad Digital Repository: http://dx.doi.org/10.5061/dryad.jr66k

## Appendix A

Visual representation of the method to define buffers and calculate habitat variables among pairs of individuals to use in MRM analyses (see Materials and methods).

In the figure below, the two black dots represent individuals for which values of habitat variables were calculated. The width of the buffer is equal to 4 km. The length of the buffer is equal to the Euclidian distance between the two individuals. Within the buffer, the proportion of the surface area covered by each habitat type is computed, providing values for pairwise habitat variables. The length of forest–field edges is also computed and divided by the buffer surface to obtain edge density (in km/km^2^).

## Appendix B

**Table A1:** Number of alleles (*k*), observed heterozygosity (*H*_O_) and expected heterozygosity (*H*_E_) for all microsatellite loci used in this study, in raccoons and striped skunks.

	Locus	*k*	*H*_O_	*H*_E_
Raccoons (*n* = 330)	PLM06	5	0.705	0.754
	PLOM2	14	0.880	0.879
	PLOM3	6	0.774	0.780
	PLM20	14	0.685	0.760
	PLO2-117	26	0.888	0.931
	PLOM15	17	0.844	0.868
	PLO2-14	27	0.811	0.879
	PLOM17	8	0.779	0.806
	PLM10	11	0.842	0.857
	PLOM20	13	0.838	0.849
Skunks (*n* = 345)	MEPH4215	7	0.588	0.620
	MEPH2216	10	0.754	0.790
	MPEH2270	19	0.881	0.896
	MEPH4273	13	0.794	0.809
	MEME84	12	0.832	0.857
	MEPH2214	15	0.841	0.848
	MEME15	9	0.733	0.756
	MEME75	13	0.852	0.869
	MEPH2219	9	0.780	0.800

## Appendix C

Testing the sensitivity of IBR analyses to variation in edge resistance values.

IBR analyses described in the Methods and Results were repeated with 10 different values of edge resistance for model 7 (see Table 2). Resistance of other habitat types was kept the same as in Table 2. For each edge resistance value (ranging between 1 and 45, all smaller than forest resistance), the model was then compared with the other six resistance models described in Table 2 with the pseudo-bootstrap method described in the Methods. The results are illustrated below for male raccoons, female raccoons, male skunks and female skunks. In three of the cases, the model with the greatest bootstrap support remains the same, regardless of the edge resistance value. For female raccoons however, for which results were not as unequivocal (see Fig. 2), using the lowest resistance value leads to model 7 being the ‘best model’, while other values lead to model 4 as the best model. Results reported in Fig. 2 used an edge resistance of 10 for model 7. Values on the y-axis are numbers of bootstrap replicates (total = 50 000), and model numbers are listed on the x-axis.
